# Development and validation of a flow state scale for healthcare professionals

**DOI:** 10.3389/fpsyg.2026.1751308

**Published:** 2026-04-01

**Authors:** Yunxia Zhong, Yuqing Zhang, Xiaohan Sun, Jun Li

**Affiliations:** 1 School of Public Health, Capital Medical University, Beijing, China; 2 Education Department, Beijing Hospital of Traditional Chinese Medicine, Capital Medical University, Beijing, China

**Keywords:** healthcare professionals, flow, scale, reliability, validity

## Abstract

**Purpose:**

Nowadays, healthcare workers generally face mental health risks such as occupational burnout, depression, and low job satisfaction, which negatively affect both their physical and mental well-being as well as organizational performance. Flow, as a positive internal motivational state, may help address these challenges. Therefore, this study aimed to develop and validate a flow state scale suitable for healthcare professionals offering a scientific and useful instrument for evaluating flow experience among healthcare workers.

**Methods:**

An initial item pool was generated based on a literature review, existing scales, and the specific nature of healthcare work. The items were then translated and back-translated by bilingual experts (Chinese and English) to ensure linguistic and conceptual equivalence. We refined the items through a two-round Delphi expert consultation (*n* = 15). All items were rated on a 5-point Likert scale. Participants were instructed to respond based on their experience during the medical service activity they had just completed. Using a convenience sampling strategy via an online platform, we collected 1,240 valid questionnaires from healthcare professionals in China, predominantly from Beijing and Fujian provinces. The sample was randomly divided into two equal parts. The first set (*n* = 620) was used for item analysis and initial reliability and validity testing to establish the factor structure. The second set (*n* = 620) was used for cross-validation.

**Results:**

The flow state scale for healthcare professionals includes 15 items measuring four dimensions of the flow experience: concentration and directional feedback, intrinsic empowerment and control, transformation of time, and loss of self-consciousness. The scale showed high internal consistency (Cronbach’s *α* from 0.813 to 0.911 for the full and subdimension scales). Confirmatory factor analysis supported the four-factor structure (*χ*^2^/*df* = 2.661, RMSEA = 0.073, GFI = 0.915, AGFI = 0.878, SRMR = 0.049, RMR = 0.037). While the AGFI value fell slightly below the 0.90 threshold for “good” fit, it exceeded the acceptable criterion of 0.85, and all other indices indicated good model fit. The final model was developed using the first subsample (12 items deleted based on modification indices) and cross-validated in an independent second subsample. The scale also demonstrated sound convergent and discriminant validity.

**Conclusion:**

The flow state scale for healthcare professionals has good reliability and validity. It can serve as an effective tool for assessing the flow state among healthcare professionals and provides a valuable reference for healthcare institutions in management practices such as personnel motivation, burnout intervention, performance management, and further service quality improvement.

## 1 Introduction

In today’s medical field, healthcare professionals widely face issues such as professional burnout (Zheng et al., 2022; Mohamed et al., 2025; Mutair et al., 2025), depression (Naser et al., 2025), low job satisfaction (Newton et al., 2004; Abate and Mekonnen, 2021; Diakos et al., 2023), and increasing work stress (Marković, 2019; Yu et al., 2024). These problems not only harm the physical and mental health of healthcare workers but also exacerbate talent loss (Medical Protection, 2025), reduce the quality of medical services (Hodkinson et al., 2022; Shin et al., 2023), and thus have a negative impact on the harmonious development of doctor-patient relationships (Zarei et al., 2015). In this context, how to maintain the emotional health and psychological well-being of healthcare professionals is no longer merely an individual concern, but has become a central priority for improving the organizational efficiency and operational stability of medical institutions. Although healthcare organizations generally adopt external intervention measures such as performance bonus, career development support, and team-building activities, their effectiveness is often limited and difficult to sustain. Consequently, researchers are gradually shifting their focus to internal psychological resources, especially those psychological constructs that can stimulate positive work experiences and high-efficacy states. As the peak experience state of deep immersion and intrinsic enjoyment, “Flow” has aroused wide attentions in the fields of organizational psychology and health services research.

The concept of “flow,” introduced by American psychologist Csikszentmihalyi in the 1970s, describes a holistic psychological state experienced when individuals are fully immersed in an activity (Csikszentmihalyi, 1975). In this subjective state of flow, people exhibit highly focused attention, a sense of unity between action and awareness, feelings of control, distortion of temporal experience, and intrinsic enjoyment of the process (Csikszentmihalyi, 1988; Nakamura and Csikszentmihalyi, 2009). The flow state is referred to by various terms—such as optimal performance, peak experience, and work immersion—all of which are commonly used as labels for this phenomenon (Jackson and Csikszentmihalyi, 1999). Based on systematic research, Csikszentmihalyi proposed nine characteristics of flow, including challenge-skill balance, action-awareness merging, clear goals, unambiguous feedback, concentration on the task at hand, sense of control, loss of self-consciousness, transformation of time, and autotelic experience (Jackson and Csikszentmihalyi, 1999). Bakker introduced flow theory into the field of organizational behavior, proposed the concept of work immersion, and constructed a three factor model of absorption, work enjoyment, and intrinsic work motivation (Bakker, 2008). It is noteworthy that there are essential differences in theoretical connotations or temporal characteristics between flow and similar constructs such as work engagement, absorption, and intrinsic motivation. Flow is a short-lived yet intense “peak experience state” that occurs during a specific task, whereas work engagement is a relatively persistent and pervasive emotional-cognitive state composed of three dimensions: vigor, dedication, and absorption (Schaufeli et al., 2002). In terms of temporal characteristics, the two correspond to instantaneity and relative persistence, respectively, and should not be confused. Absorption is a subdimension of work-related flow (Bakker, 2008) or work engagement (Schaufeli et al., 2002), rather than an independent construct. Absorption refers only to a state of highly focused attention, which is also context-dependent and instantaneous, but lacks the multidimensional experiential integrity of flow. Intrinsic motivation is a motivational orientation (Ryan and Deci, 2000) that drives behavior, while flow is a subjective experiential state during the behavioral process. Intrinsic motivation stems from self-determination (Deci and Ryan, 1985), which is a pre-driving force for behavior and one of the important preconditions for the generation of flow. As a core concept of positive psychology, flow has been proven to play an important role in improving job performance (Engeser and Rheinberg, 2008; Peifer and Wolters, 2021; Chatterjee, 2023) and promoting positive emotional experiences (Keller and Landhäußer, 2012; Mao et al., 2020) in multiple fields. In the medical field, the flow state is expected to become a key intrinsic motivation factor, helping healthcare workers balance work challenges with personal skills and gain intrinsic satisfaction from their work, so as to provide a new perspective for alleviating burnout, improving work engagement and medical service quality.

Although the flow theory has been widely explored across various fields, existing measurement tools are still mainly designed around sports (Stavrou et al., 2015; Norsworthy et al., 2018; Yarayan et al., 2025), education (Egbert, 2003; Shernoff et al., 2003; Shernoff et al., 2014), or online games (Sweetser and Wyeth, 2005; Perttula et al., 2017), lacking sufficient consideration for the particularities of the medical professional environment. The most representative flow measurement tools currently include the Flow State Scale (FSS) developed by Jackson and Marsh, (1996) and its subsequent revised versions, which are based on a nine dimensional theoretical model and have good psychometric characteristics. However, the item design and situational setting are mostly derived from sports or general task environments. Directly transplanting such tools to the medical scenario may face the following psychometric risks: First, differential item functioning. Healthcare work is usually considered to be highly professional, complicated, ethically rigid (Breen et al., 2010), and requires a large amount of emotional labor (Chen et al., 2022). The same items may have different understandings and interpretations between healthcare professionals and other professional groups, leading to cross-group comparison biases. Second, the dimensional structure may be incompatible. Jackson’s nine-factor model was established within the context of sports psychology, and its dimensional distinguishability relies on the background characteristics of relatively clear sports tasks and single roles. However, healthcare work has complex characteristics such as multitasking (Yen et al., 2016), close team collaboration (Chien et al., 2012), and serious responsibility consequences (Castillo et al., 2021), which may cause the dimensions of flow to merge and reorganize at the experiential level, rather than simply maintaining the original nine-dimensional structure. Therefore, directly applying existing general flow scales may not only result in poor model fit due to structural incompatibility but may also obscure the true organization of flow experiences in medical contexts, thereby limiting the practical application value and research depth of the theory in healthcare organizational management. At present, international research on the work status of healthcare workers remains predominantly focused on negative indicators such as stress(Feleke et al., 2025), burnout(Uccheddu et al., 2025), compassion fatigue(Ondrejková and Halamová, 2022), and job satisfaction(Cantarelli et al., 2023). Research on positive psychological states, particularly flow, is still in its preliminary stages. A few prospective studies suggest that flow experiences may have a protective effect on the well-being of healthcare workers (Akman and Çatar, 2019). However, due to the lack of standardized and contextually adapted assessment tools suitable for this group, it is difficult for relevant studies to systematically explore the causes and consequences, mechanism and effective organizational intervention strategies of healthcare professionals’ flow experience. Consequently, there is an urgent need to develop a flow assessment tool with professional relevance and psychometric robustness to advance research in healthcare contexts.

Therefore, the purpose of this study is to develop and validate a flow state scale suitable for healthcare professionals. Based on the “flow state scale” compiled by Jackson and Marsh (1996) and Liu’s (2009) revised Chinese version, this research will integrate the specific context and professional characteristics of medical work. Through multiple phases, including a systematic literature review, Delphi expert consultation, item analysis, and exploratory and confirmatory factor analyses, the study seeks to construct a multidimensional assessment tool with good reliability, validity, and practicality. This study presumes that in confirmatory factor analysis, the factor structure of the scale should meet the following acceptable model fit criteria: *χ*^2^/*df* ≤ 3.00, RMSEA ≤ 0.08, SRMR ≤ 0.05, CFI ≥ 0.90, AGFI ≥ 0.85 (Kline, 2016; Ntumi et al., 2025). This scale will be able to yield not only an applicable instrument for the scientific assessment of flow states among healthcare professionals but also a practical reference for healthcare institutions to adjust their human resource management with a positive psychology view, design intrinsic motivation approach and implement early prevention and psychological interventions against occupational burnout.

## 2 Materials and methods

**Figure 1:**
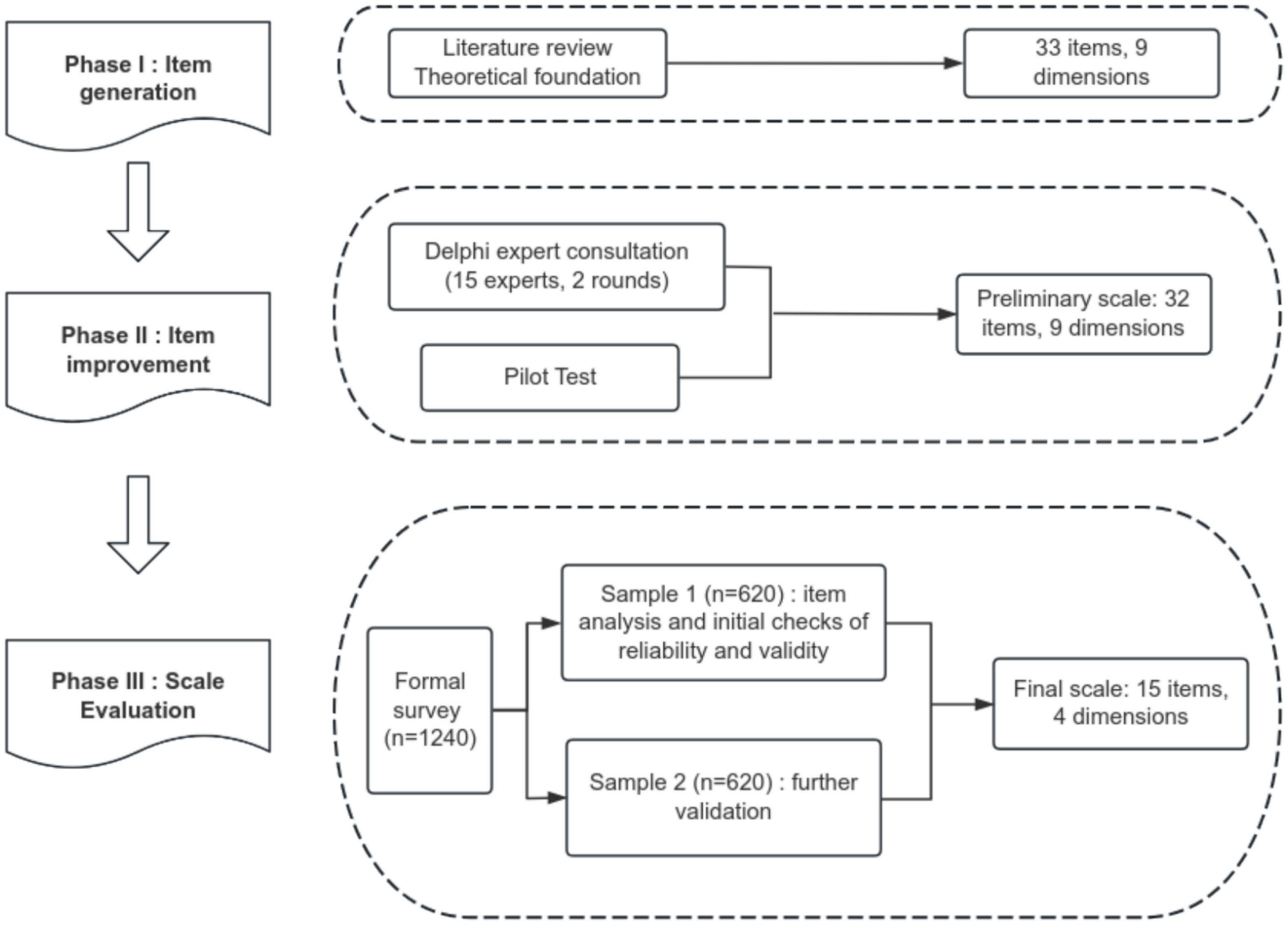
The development and validation process of the flow state scale for healthcare professionals.

The development and validation of the scale were adopted the approach proposed by DeVellis (1991) and Hinkin (1995). The approach includes three steps: item generation; item improvement; and scale evaluation. The study was approved by the Medical Ethics Committee of Capital Medical University (Ethics Code: Z2022SY019). Figure 1 shows the development and validation process of the scale.

### 2.1 Phase I: item generation

A comprehensive search of the literature was performed using the CNKI, VIP, WanFang, and Web of Science databases. The search scope covers publications up to November 5th, 2024. The search strategy combined three conceptual domains using Boolean operators: (1) flow-related terms: (“flow” OR “flow state” OR “work immersion” OR “flow theory” OR “optimal experience”); (2) healthcare professional terms: (“healthcare professional” OR “healthcare worker” OR “medical staff” OR “physician” OR “nurse” OR “clinician”); (3) measurement tool terms: (“scale” OR “questionnaire” OR “assessment” OR “measure”). The full search string for Web of Science was: TS = ((“flow” OR “flow state” OR “work immersion” OR “flow theory”) AND (“healthcare professional” OR “healthcare worker” OR “medical staff” OR “physician” OR “nurse”) AND (“scale” OR “questionnaire” OR “assessment” OR “measure”)). Equivalent Chinese terms were used for Chinese databases. Inclusion criteria: (a) focusing on the core constructs of flow state, optimal experience, or work-related flow; (b) using quantitative measurement tools (scales, questionnaires); (c) published in peer-reviewed journals or Chinese/English academic dissertations. Exclusion criteria: (a) measuring other constructs rather than flow; (b) reviews, editorials, conference abstracts, without original scale development or validation data; (c) full text not accessible. The literature screening process followed the PRISMA guidelines and was independently conducted by two researchers. Disagreements were resolved through discussion or adjudication by a third researcher.

Based on a systematic literature review and building upon Jackson’s Flow State Scale and its Chinese version adapted by Weina Liu, the initial item pool was developed to reflect the specific working environment of healthcare staff. The items were translated and back-translated by two bilingual experts to ensure consistency in language expression and conceptual connotation. Subsequently, three senior healthcare staff from different positions (including doctors, nurses, and medical technicians) were invited for cognitive interviews to assess the clarity, comprehensibility, and degree of reflection of the items on flow experience in the process of medical services. Based on the interview feedback, some items were linguistically adjusted to better fit the medical work context. This preliminary version included 33 items across nine dimensions: Challenge-Skill Balance, Action-Awareness Merging, Clear Goals, Unambiguous Feedback, Concentration on the Task at Hand, Sense of Control, Loss of Self-Consciousness, Transformation of Time, and Autotelic Experience (for the mapping table from theoretical dimensions to initial healthcare-contextualized entries, please refer to Supplementary material).

### 2.2 Phase II: item improvement

The initial items were further optimized after two rounds of Delphi expert consultation via email from May to July 2024. The panel consisted of 15 medical experts and senior healthcare workers from different institutions in Beijing. The inclusion criteria for experts were: ①from different medical institutions and different specialties (including surgery, internal medicine, pediatrics, obstetrics and gynecology, psychology, etc.), with different professional titles (including junior, intermediate, associate senior, and senior), and had at least 2 years of clinical experience in their current professional field; ② bachelor’s degree or above; ③ voluntarily participated in this study. The average working years of the consulted experts was 16.5 years, and their work directions involved surgery (3), internal medicine (2), psychology (3), healthcare management (2), nursing (1), traditional chinese medicine (1), obstetrics and gynecology (1), pediatrics (1), and other fields (1). The expert authority coefficient (Cr) was 0.815, indicating that the expert panel possessed high authority and professional representativeness. Experts were asked to rate each item on importance and operability as well as sensitivity (Hsu and Sandford, 2007). Item extraction was performed by using a threshold method. The mean, full-score ratio and coefficient of variation of the importance score were calculated (Gore et al., 2021). The criteria for determining the threshold values were as follows: for the full score ratio and the mean, the threshold was calculated as “threshold = mean - standard deviation,” and items with scores above the threshold were retained; for the coefficient of variation (CV), the threshold was calculated as “threshold = mean + standard deviation,” and items with scores below the threshold were retained. To prevent important indicators from being removed, items that failed to meet the requirements on all three measurement scales were eliminated. For example, in the first round of expert consultation, the item “I was not concerned about my self-presentation in medical services” had a mean of 3.7 (threshold = 4.08), a full-score ratio of 0.33 (threshold = 0.46), and a CV of 0.29 (threshold = 0.26), all of which did not meet the standards, so it was deleted. For items where one or two measures did not meet the requirements, decisions were made through expert discussion based on principles such as comprehensiveness and feasibility. At the same time, the modification suggestions proposed by experts were fully considered in the selection of indicators. The effective response rate for both consultation rounds was 100.00%. One item was deleted and ten items were revised according to experts’ suggestions after the first round of consultation. Eight more items were revised after the second round.

Kendall’s coefficient of concordance was 0.14 (*p* < 0.001) in round 1 and 0.22 (*p* < 0001) in round 2, indicating a tendency toward consensus. In Delphi studies focusing on multidimensional and subjective constructs such as flow, a moderate W value is not uncommon due to the diverse backgrounds of experts (Su et al., 2025; Wang et al., 2025), reflecting the process of integrating different professional perspectives. This study adopted multiple indicators to comprehensively assess consensus achievement: (1) Central tendency of opinions: the mean importance score of items increased from 4.08 in the first round to 4.21 in the second round (on a 5-point scale); (2) Convergence of opinions: the coefficient of variation (CV) for the importance of all items decreased to below 0.25 in the second round (most <0.20), meeting the criteria for good consensus (Sun and Zhang, 2025); (3) Stability of opinions: Based on the method proposed by Bhandari et al., (2020), we evaluated the stability of expert ratings between the two rounds. Wilcoxon signed-rank tests were conducted on the 32 items that remained unchanged across both rounds. To account for multiple comparisons, the Bonferroni correction was applied (Staffa and Zurakowski, 2020). The results indicated no significant differences in expert scores between rounds. Based on the increase in mean scores, the CV values meeting the standard, and statistical stability, it was comprehensively determined that expert opinions had reached consensus. Therefore, the consultation was terminated. The final preliminary scale contained 32 items in 9 dimensions.

Next, we carried out a pilot test with 70 individuals through an electronic questionnaire. This was used to estimate the time to complete the questionnaire and to assess the clarity and comprehensibility of the items. The pilot test employed the same screening criteria as the formal survey, recruiting healthcare workers through convenient sampling, ultimately collecting 65 valid questionnaires (with a valid response rate of 92.86%). Participants came from hospitals of different levels (from primary to tertiary hospitals) and included various professional categories such as doctors, nurses, and pharmacists, providing a certain level of occupational and institutional representativeness. The results of the pilot scale showed that its internal consistency was good (Cronbach’s *α* = 0.96), and it was suitable to perform factor analysis (KMO = 0.84, *p* < 0.05) and the participants considered the items easy to understand, comprehensive and unambiguous (Al-Rousan et al., 2025).

### 2.3 Phase III: scale evaluation

#### 2.3.1 Participants and data collection

The formal survey was conducted from March to May 2025. Considering the practical difficulties of reaching busy healthcare professionals nationwide through purely random sampling, this study adopted a convenience sampling strategy, conducted via the widely used Chinese online survey platform “Wenjuanxing.” This method facilitated efficient recruitment across different geographical regions and types of institutions. The study included healthcare professionals working in various clinical departments across different levels of medical institutions in China. The inclusion criteria were: (1) working in various medical institutions and departments, such as surgery, internal medicine, pediatrics, obstetrics and Gynecology, anesthesiology and other related fields, and covering all professional titles from junior to senior; (2) having at least 2 years of continuous clinical work experience in the current professional field. This criterion ensured that participants had passed the initial training period and developed a stable understanding of their work routines and challenges, thereby enabling them to provide meaningful reports on their flow states during medical services; (3) voluntary participation in this study (Yue et al., 2022).

Due to the potential risks of online convenience sampling, such as self-selection, profession imbalance, and platform-specific biases, we implemented the following strategies to mitigate these potential biases and enhance sample representativeness as much as possible: (1) During the recruitment phase, although we did not set strict numerical quotas, we established clear stratified recruitment objectives. We stratified the target population by hospital level (Grassroots/Unrated/Primary/Secondary/Tertiary) and core professional categories (Physician/nurse/Pharmacist/Medical technician/other). We expanded the distribution scope of the questionnaire within each stratum by contacting health administrative departments, hospital management departments, and social media channels targeting specific occupational groups in different regions, striving to ensure that the sample covers all preset levels. (2) The collected questionnaires underwent a multi-step cleaning process before analysis. Responses with completion times less than 180 s were marked. A total of 58 questionnaires (4.26%) were eliminated based on this time threshold. We screened for contradictory responses to questions with inherent logical connections in the same dimension (e.g., “During the medical service process, I did not care how patients or colleagues viewed me” and “During the medical service process, I was not worried about how patients or colleagues might view me”), and 34 questionnaires (2.5%) were excluded due to obvious logical contradictions. Responses exhibiting uniform extreme values (such as all selecting 1) in long-sequence questions were reviewed, and after comprehensive judgment combined with response time, 28 questionnaires (2.06%) were eliminated through this step. After applying the above cleaning rules, out of the initially collected 1,360 questionnaires, 1,240 valid questionnaires were ultimately retained, with an effective response rate of 91.18%.

#### 2.3.2 Data analysis

This study used the random case selection function of SPSS 26.0 to randomly divide all 1,240 valid questionnaires into two sub-samples. Employing the simple random sampling method without replacement, the entire process was automatically completed by the software to ensure that each case had an equal probability of being selected into either sub-sample, thereby minimizing selection bias. Sample 1 (*n* = 620) was used for item analysis and initial reliability and validity testing to establish the factor structure. Sample 2 (*n* = 620) served as an independent dataset for cross-validation to confirm the stability of the factor structure obtained in Sample 1 (Zhang and Li, 2021). All statistical analyses were conducted using SPSS Statistics (Version 26.0) and AMOS (Version 26.0).

Before conducting factor analysis, we evaluated the statistical assumptions. The Kolmogorov–Smirnov test and Shapiro–Wilk test were used to assess the normality of item distribution, and the results indicated that the sample was non-normal. Therefore, exploratory factor analysis employed principal axis factoring, which does not require multivariate normality, combined with Promax rotation. The Kaiser–Meyer–Olkin (KMO) measure and Bartlett’s test were used to evaluate sampling adequacy and factor analyzability. Item analysis was conducted using the critical ratio method and corrected item-total correlations. Multicollinearity diagnostics showed that the variance inflation factor for all items was less than 5 and the tolerance was greater than 0.2, indicating no severe redundancy. In exploratory factor analysis, items with factor loadings less than 0.5 or with cross-loadings were considered for deletion. EFA was repeated after each deletion to ensure the stability of the factor structure, and the iteration continued until a clear factor structure was obtained. CFA was conducted based on structural equation modeling to test the goodness-of fit of the proposed factor structure. Model fit was evaluated using several indices, including the chi square to degrees of freedom ratio (*χ*^2^/*df*), Root Mean Square Error of Approximation (RMSEA), Comparative Fit Index (CFI), Tucker–Lewis Index (TLI), Standardized Root Mean Square Residual (SRMR), and Root Mean Square Residual (RMR). The model modification followed the following process: First, factor loadings were examined. Items with standardized factor loadings below 0.5 were considered for deletion, with only the item having the lowest loading deleted at each iteration before re-running the model. Next, the model fit indices were checked and the process stopped if they met the criteria. Finally, the modification indices (MI) were examined and the largest MI value was identified. A high MI usually indicated redundancy between two items within the same factor. Therefore, only items belonging to the same latent variable were considered, and error correlations across factors were not allowed, as this approach lacked theoretical justification and might alter the factor structure. After removing the item that caused the highest MI, the model was rerun (Awang, 2015). This iterative process was repeated until all fit indices reached an acceptable level. Construct validity was tested by both the convergent validity and discriminant validity. The internal consistency of the entire scale and each subdimension was evaluated using Cronbach’s *α* coefficient.

## 3 Results

### 3.1 Participants characteristics

The analysis included 1,240 valid questionnaires. Female respondents accounted for 69.35% (*n* = 860) of the sample, while males made up 30.32% (*n* = 376). The age distribution was primarily composed of young and middle-aged adults: 23.15% were 25 years or younger, 36.94% were between 26 and 35 years old, 23.39% were aged 36 to 45, and 16.53% were 46 or older. In terms of education, the largest group held a bachelor’s degree (53.23%, *n* = 660). Regarding work experience, those with more than 10 years of service formed the largest segment (48.55%, *n* = 602). The majority of respondents worked in tertiary hospitals (61.13%, *n* = 758). By profession, physicians were the most represented group (63.55%, *n* = 788). In departmental distribution, internal medicine (23.06%), general practice (24.03%), and other specialties (19.76%) had the highest representation. The detailed demographic characteristics are presented in Table 1.

**Table 1:** Demographic characteristics of participants (*n* = 1,240).

Characteristics	Categories	N	Percentage (%)
Gender	Male	376	30.32
	Female	860	69.35
	Other genders	4	0.32
Age	25 or below	287	23.15
	26–35	458	36.94
	36–45	290	23.39
	46 or above	205	16.53
Education level	Associate degree or below	213	17.18
	Bachelor’s degree	660	53.23
	Master’s degree	256	20.65
	Doctoral degree	111	8.95
Work experience	≤6 years	475	38.31
	7–9 years	163	13.15
	≥10 years	602	48.55
Hospital level	Grassroots medical institutions	53	4.27
	Unrated institutions	37	2.98
	Primary hospital	270	21.77
	Secondary hospital	122	9.84
	Tertiary hospital	758	61.13
Professional category	Physician	788	63.55
	Nurse	257	20.73
	Pharmacist	38	3.06
	Medical technician	100	8.06
	Other	57	4.60
Department	Internal medicine	286	23.06
	Surgery	153	12.34
	Pediatrics	42	3.39
	Obstetrics and gynecology	48	3.87
	Traditional Chinese medicine	40	3.23
	Anesthesiology	23	1.85
	Medical technology departments	105	8.47
	General practitioner	298	24.03
	Other	245	19.76

### 3.2 Item analysis

A systematic item analysis was conducted on Sample 1 (*n* = 620). The Kolmogorov–Smirnov and Shapiro–Wilk tests indicated that all items were non-normally distributed (*p* < 0.001). The corrected item-total correlation (CITC) values ranged from 0.336 to 0.795, all above 0.3, suggesting good internal consistency of the items within their respective dimensions (Frerichs et al., 2025). Ceiling/floor effect evaluation showed that most items had extreme response rates below 30%, indicating a good range of responses. However, five items (Q3, Q4, Q12, Q13, and Q32) showed slight ceiling effects, with selection rates between 30.5 and 35.8%. Considering that the survey sample had a high proportion of senior healthcare staff (48.55% with ≥10 years of work experience), this slight bias may reflect the genuinely high level of flow experience in this subgroup. Independent samples t-tests on the extreme groups (the highest and lowest 27% of total scores) revealed that the score differences for all measurement items between the two groups reached statistical significance (*p* < 0.05 and *t*-value greater than 3) (Zetriuslita et al., 2021). Finally, variance inflation factor (VIF) and tolerance were used for multicollinearity diagnosis. The results showed that the VIF for all items was less than 5, and the tolerance was greater than 0.2, indicating no severe redundancy among the items (Hair, 2009). Based on these indicators, we decided to retain all 32 items for subsequent exploratory factor analysis.

### 3.3 Validity analysis

The scale’s content validity was supported by its grounding in existing well-established instruments, contextual adaptation for medical workers, and systematic optimization through Delphi consultations and a pre-test (Song et al., 2018). For structural validity evaluation, this study split sample one and sample two in half, performing exploratory and confirmatory factor analysis, respectively.

### 3.4 Exploratory factor analysis

KMO value greater than 0.7 usually indicates good scale validity (Shen et al., 2025). In the initial analysis, the KMO value reached 0.956, well above the 0.7 threshold. Bartlett’s test of sphericity also showed significant results (*p* < 0.001), meaning that the data are suitable for factor analysis. As the first subsample is not normally distributed, we analyzed the factor structure with Principal Axis Factoring (PAF), and rotated it with a Promax rotation (Fabrigar et al., 1999) based on the assumption that dimensions are correlated within the flow construct (Shanxi Medical University, 2013).

To determine the optimal number of factors to retain, we initially considered using parallel analysis. However, preliminary analysis revealed that the results of parallel analysis were sensitive to sample size, with the recommended number of factors increasing from 3 to 4 as the sample size expanded. In contrast, the traditional method of factor extraction using eigenvalues and the scree plot yielded relatively stable results across different sample sizes. Therefore, the latter approach was prioritized, and items were deleted step by step according to the criteria of factor loadings below 0.5 (Osborne and Costello, 2009) or the presence of cross-loadings (defined as a loading of 0.4 or higher on two or more factors) (Lin and Wu, 2014). The analysis process was rerun after each removal until a good result was obtained. Through multiple rounds of EFA, items Q1, Q2, Q11, Q20, Q24 were deleted, finally retaining 27 items, yielding 4 common factors. The eigenvalues of the first four factors were 13.658, 2.109, 1.676, and 1.105, respectively, cumulatively explaining 63.29% of the total variance. Communalities ranged from 0.420 to 0.748, all exceeding the recommended threshold of 0.4 (Hair, 2009), indicating that each item was sufficiently explained by the common factors. The overall KMO value was 0.952, *p* < 0.001. The factor correlation matrix showed that the correlation coefficients among the four factors ranged from 0.429 to 0.767. These moderate to strong correlations confirmed that the factors were interrelated yet independent constructs, justifying the use of the Promax rotation method. Factor 1 included 12 items, mainly composed of Clear Goals, Unambiguous Feedback, and Concentration on the Task at Hand from the initial scale, and was named “Concentration and Directional Feedback”. Factor 2 included 8 items, mainly composed of Autotelic Experience, Challenge-Skill Balance, and Sense of Control from the initial scale, and was named “Intrinsic Empowerment and Control”. Factor 3 included 4 items, composed of the Transformation of Time from the initial scale, and was still named “Transformation of Time”. Factor 4 included 3 items, composed of the Loss of Self-Consciousness from the initial scale, and was named “Loss of Self-Consciousness” (Table 2).

**Table 2:** Factor loadings from exploratory factor analysis (sample 1).

Dimension	Item	Factor 1	Factor 2	Factor 3	Factor 4
Concentration and directional feedback	Q3. During the medical service, I knew clearly what I wanted to do.	0.827			
	Q4. I was indeed very clear about how I was performing in the medical service.	0.896			
	Q5. My attention was completely focused on the current medical service work.	0.874			
	Q6. I felt in control of my medical service activities.	0.695			
	Q12. I was clearly aware of my medical service duties and goals.	0.812			
	Q13. I understood my performance in the medical service.	0.833			
	Q14. I could concentrate on the current medical service work without any effort.	0.513			
	Q21. My goals for the medical service were clearly defined.	0.701			
	Q22. During the medical service task, I was fully aware of how I was performing.	0.806			
	Q23. I was fully absorbed in every detail of the current medical service work.	0.647			
	Q29. I could judge my performance based on the medical service task I was completing.	0.614			
	Q30. I was fully immersed in the current medical service work.	0.640			
Intrinsic empowerment and control	Q9. I greatly enjoyed the moments during the medical service.		0.674		
	Q10. My medical service skills matched the high challenges of the current situation.		0.620		
	Q15. I felt I could (or was able to) completely control every aspect of the current medical service activity.		0.542		
	Q18. I loved this feeling of providing medical services and wanted to experience it again.		0.935		
	Q19. I felt my ability was sufficient to meet the high demands of the current medical service situation.		0.575		
	Q27. The medical service experience made me feel very good.		0.972		
	Q28. The medical service challenges and my professional skills were both at equally high levels.		0.541		
	Q32. The experience of providing medical service to patients was the best form of encouragement.		0.574		
Transformation of time	Q8. During the medical service, time seemed to change.			0.821	
	Q17. During the current medical service activity, time passed differently than usual.			0.761	
	Q26. During the medical service, I lost the normal sense of time.			0.669	
	Q31. I felt that time passed more quickly during the medical service than usual.			0.531	
Loss of self-consciousness	Q7. During the medical service process, I did not care how patients or colleagues viewed me.				0.766
	Q16. During the medical service process, I was not concerned about how others might evaluate my medical service behavior.				0.843
	Q25. During the medical service process, I was not worried about how patients or colleagues might view me.				0.753

### 3.5 Confirmatory factor analysis

**Figure 2:**
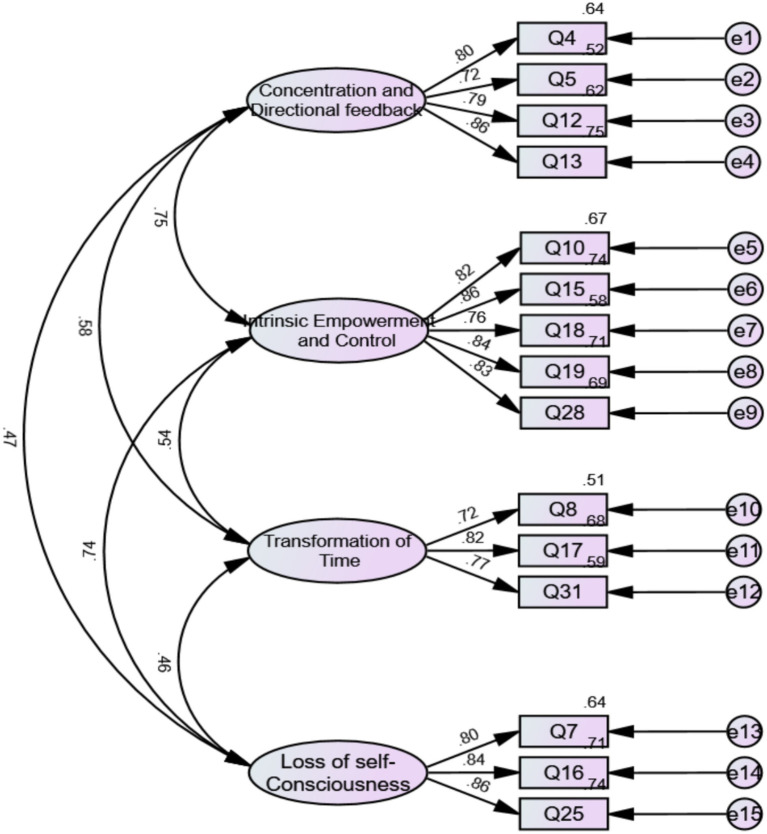
CFA model with standardized factor loadings (sample 1).

The CFA was conducted using AMOS version 26.0. Following each model modification, the fit indices were evaluated against established criteria. When the indicators suggested an inadequate fit, further optimization was undertaken until the model achieves all requisite standards. Finally, through CFA, items Q3, Q6, Q9, Q14, Q21, Q22, Q23, Q26, Q27, Q29, Q30, Q32 were deleted. The final model comprised 15 items across four dimensions (Table 3). The CFA model with standardized factor loadings is presented in Figure 2.

**Table 3:** Final item distribution across dimensions.

Dimension	Final items	Item wording
Concentration and directional feedback	4	Q4. I was indeed very clear about how I was performing in the medical service. Q5. My attention was completely focused on the current medical service work. Q12. I was clearly aware of my medical service duties and goals. Q13. I understood my performance in the medical service.
Intrinsic empowerment and control	5	Q10. My medical service skills matched the high challenges of the current situation. Q15. I felt I could (or was able to) completely control every aspect of the current medical service activity. Q18. I loved this feeling of providing medical services and wanted to experience it again. Q19. I felt my ability was sufficient to meet the high demands of the current medical service situation. Q28. The medical service challenges and my professional skills were both at equally high levels.
Loss of self-consciousness	3	Q7. During the medical service process, I did not care how patients or colleagues viewed me. Q16. During the medical service process, I was not concerned about how others might evaluate my medical service behavior. Q25. During the medical service process, I was not worried about how patients or colleagues might view me.
Transformation of time	3	Q8. During the medical service, time seemed to change. Q17. During the current medical service activity, time passed differently than usual. Q31. I felt that time passed more quickly during the medical service than usual.

The construct validity of the model was supported by goodness-of-fit indices: *χ*^2^/*df* = 2.596, RMSEA = 0.072, GFI = 0.916, AGFI = 0.880, CFI = 0.955, IFI = 0.955, TLI = 0.944, SRMR = 0.047, and RMR = 0.047. Although the AGFI value of this study met the acceptable standard (≥0.85), it was slightly below the good fit threshold of 0.90 (Schermelleh-Engel et al., 2003). This may suggest a certain risk of model specification error. However, considering that AGFI is sensitive to sample size and model complexity (Sharma et al., 2005), and other fit indices (CFI, TLI, RMSEA, SRMR) all met or exceeded the recommended standards (Shi and Maydeu-Olivares, 2020), we consider the overall model fit to be acceptable. A detailed summary of the retention and deletion of the initial nine flow dimensions after CFA can be found in Supplementary material.

### 3.6 Convergent and discriminant validity

Convergent validity was supported by average variance extracted (AVE) values exceeding 0.5 and composite reliability (CR) values above 0.7 for all dimensions (Hair et al., 2010). The results for discriminant validity showed that the square root of the AVE (minimum value) was greater than the maximum value of all correlation coefficients (Fornell and Larcker, 1981) (Table 4). To provide a more rigorous test of discriminant validity, we further calculated the heterotrait-monotrait ratio (HTMT) and estimated its 95% confidence interval using the bias-corrected bootstrap method (5,000 resamples). The results showed that all HTMT values in Sample 1 were below the threshold of 0.90, ranging from 0.456 to 0.769, and their 95% confidence intervals (the upper bounds of the 95% one-tailed confidence intervals) also did not exceed 0.90 (Henseler et al., 2015). Based on this, the scale can be considered to have good discriminant validity. Detailed HTMT matrix and confidence interval results are presented in Supplementary Tables S2, S3.

**Table 4:** Convergent and discriminant validity: composite reliability (CR), average variance extracted (AVE), and inter-construct correlations (sample 1).

Dimension	CR	AVE	1	2	3	4
1. Concentration and directional feedback	0.873	0.633	0.795			
2. Intrinsic empowerment and control	0.913	0.677	0.754	0.823		
3. Transformation of time	0.813	0.592	0.581	0.542	0.770	
4. Loss of self-consciousness	0.873	0.697	0.469	0.737	0.460	0.835

### 3.7 Reliability analysis

The reliability of the scale was assessed using Cronbach’s *α*. In sample 1, the values for the four dimensions ranged from 0.804 to 0.908, while the overall *α* coefficient reached 0.922 (Table 5). Furthermore, all items demonstrated a corrected item-total correlation (CITC) above the 0.3 threshold. The “Cronbach’s Alpha if Item Deleted” value for each one was also lower than the overall scale’s coefficient (Akbari et al., 2024), confirming the internal consistency.

**Table 5:** Reliability analysis: Cronbach’s *α* coefficients (sample 1).

Dimension	Cronbach’s *α* coefficient	Number of items
Concentration and directional feedback	0.874	4
Intrinsic empowerment and control	0.908	5
Loss of self-consciousness	0.870	3
Transformation of time	0.804	3
Overall	0.922	15

### 3.8 Cross-validation with sample 2

To cross-validate the four-factor structure established in Sample 1, we first conducted an exploratory factor analysis (EFA) on the halved Sample 2. As shown in Table 6, the EFA results clearly replicated the four-factor structure, with all items loading on the expected factors (loadings > 0.5) and collectively explaining 68.14% of the total variance (KMO = 0.915, *p* < 0.001). Replicating this structure in an independent sample through EFA provides preliminary evidence for the stability of the scale’s structure.

**Table 6:** Factor loadings from exploratory factor analysis (sample 2).

Dimension	Item number	Factor			
		1	2	3	4
Concentration and directional feedback	Q4	0.858			
	Q5	0.812			
	Q12	0.815			
	Q13	0.776			
Intrinsic empowerment and control	Q10		0.710		
	Q15		0.563		
	Q18		0.852		
	Q19		0.898		
	Q28		0.754		
Transformation of time	Q8				0.709
	Q17				0.725
	Q31				0.503
Loss of self-consciousness	Q7			0.744	
	Q16			0.919	
	Q25			0.791	

**Figure 3:**
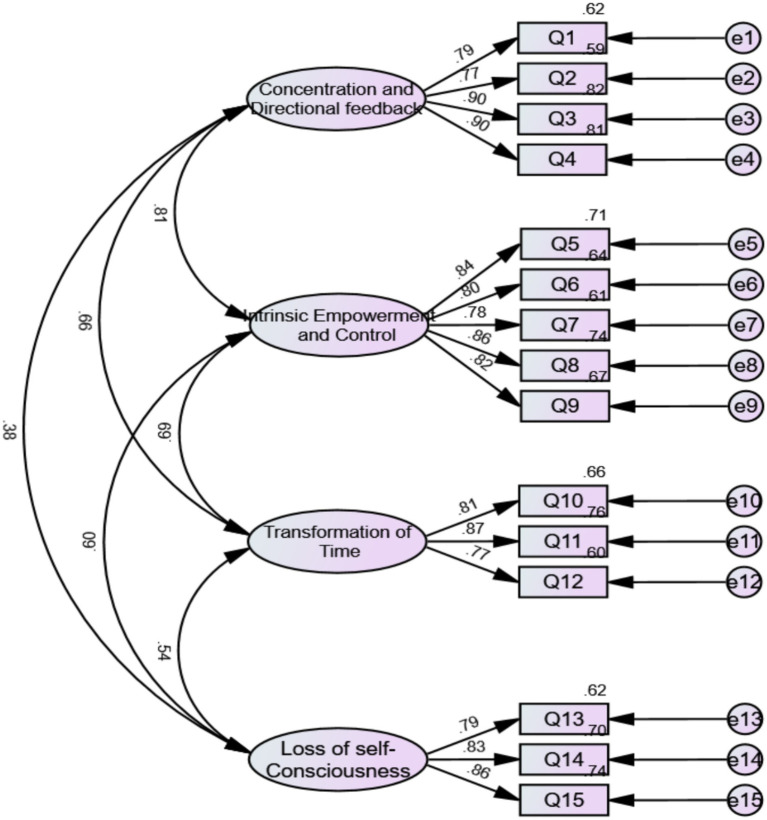
CFA model with standardized factor loadings (sample 2). The items in Figure 3 correspond to those in Figure 2, and have been renumbered for ease of reading. For the complete correspondence between the new item numbers and the original item numbers, please refer to Supplementary material.

After EFA, we conducted a confirmatory factor analysis (CFA) on the other half of the sample 2 to formally test the model fit. The CFA model with standardized factor loadings for sample 2 is shown in Figure 3. All factor loadings exceeded the recommended threshold of 0.5, indicating strong relationships between the observed items and their respective latent constructs. The model fit indices for Sample 2 were *χ*^2^/*df* = 2.661, RMSEA = 0.073, GFI = 0.915, AGFI = 0.878, CFI = 0.958, IFI = 0.959, TLI = 0.948, SRMR = 0.049, RMR = 0.037, indicating good model fit. Convergent and discriminant validity were again supported (Tables 7). The results of the HTMT matrix and confidence intervals are presented in Supplementary Tables S4, S5.

**Table 7:** Convergent and discriminant validity: composite reliability (CR), average variance extracted (AVE), and inter-construct correlations (sample 2).

Dimension	CR	AVE	1	2	3	4
1. Concentration and directional feedback	0.907	0.711	0.843			
2. Intrinsic empowerment and control	0.912	0.674	0.806	0.821		
3. Transformation of time	0.859	0.671	0.660	0.690	0.819	
4. Loss of self-consciousness	0.868	0.686	0.380	0.604	0.540	0.829

As presented in Table 8, the reliability analysis for Sample 2 yielded satisfactory results. All four dimensions achieved Cronbach’s *α* scores above 0.8, and the full scale obtained a coefficient of 0.909, attesting to its good reliability.

**Table 8:** Reliability analysis: Cronbach’s *α* coefficients (sample 2).

Dimension	Cronbach’s *α* coefficient	Number of items
Concentration and directional feedback	0.909	4
Intrinsic empowerment and control	0.911	5
Loss of self-consciousness	0.867	3
Transformation of time	0.813	3
Overall	0.909	15

## 4 Discussion

This study developed a Flow State Scale for healthcare professionals by integrating literature analysis, Delphi expert consultation, and adaptations from existing scales. The final instrument comprises 15 items across four factors: Concentration and Directional Feedback, Intrinsic Empowerment and Control, Loss of Self-Consciousness, and Transformation of Time. Confirmatory factor analysis confirmed a good model fit (*χ*^2^/*df* = 2.661, RMSEA = 0.073, GFI = 0.915, CFI = 0.958, SRMR = 0.049, RMR = 0.037). The scale showed strong reliability, with Cronbach’s *α* values between 0.813 and 0.911 for both the overall instrument and its subdimensions. All convergent validity indicators have met the standard thresholds, with AVE above 0.5 and CR above 0.7, while discriminant validity also met psychometric requirements.

The core finding of this study is reflected in the factor structure of the scale. The nine dimensions set initially based on Jackson’s et al. flow state scale framework naturally aggregated into four more general comprehensive factors in the process of exploratory factor analysis. This structural change not only conforms to the principle of statistical simplicity, but also reveals at the conceptual level that in the high-pressure and complex medical working environment, the flow experience of healthcare workers presents a highly integrated psychological state. To further verify the robustness of the four-factor structure, we conducted a comparative analysis of alternative models in Sample 2, including a single-factor model, two-factor model, three-factor model, and second-order factor model. The results showed that the four-factor model performed the best on all fit indices (see Supplementary Table S6, Supplementary material), indicating that this structure is statistically more reasonable and more explanatory.

The Concentration and Directional Feedback dimension integrates Clear Goals, Unambiguous Feedback, and Concentration on the Task at Hand. This integration shows that for healthcare professionals, the objectives of clinical work (diagnoses and treatment plans), immediate feedback about the patient (vital signs and change of symptoms), and the necessary high level of concentration are inseparable psychological and behavioral processes. The informational immediacy of the work of a medical practitioner and its goal-oriented nature (Boeykens et al., 2022) offers constant directional guidance, thus creating and holding a state of deep focus.

The dimension of Intrinsic Empowerment and Control integrates Challenge-Skill Balance, Sense of Control, and Autotelic Experience. When healthcare professionals are able to successfully respond to clinical challenges through their professional abilities, it generates not only a sense of control over external situations, but also an inner motivation and satisfaction resulting from exercising their abilities and solving problems. This kind of intrinsic reward inspired by professional competence may become a key psychological resource in the medical field with prominent occupational stress and high risk of burnout, helping practitioners maintain work enthusiasm and psychological resilience. In other words, the real empowerment may not come from the complete control of the external environment, but from the internal satisfaction and sense of achievement caused by the dynamic balance between personal ability and work challenges.

Furthermore, the classic flow characteristics of Transformation of Time and Loss of Self-Consciousness were still preserved as stable dimensions in this study. This indicates that even when practitioners are immersed in high-pressure, complex, and dynamic medical situations, they can still experience these iconic states when fully engaged. This discovery also confirms the cross-situational consistency of these core flow features in professional and social life, and supports the effectiveness of the scale in capturing states of deep engagement.

In this study, Action-Awareness Merging did not emerge as an independent dimension. In the exploratory factor analysis of Sample 1, the items corresponding to this dimension were progressively deleted due to factor loadings below 0.5. From a conceptual perspective, this may be related to the cognitive complexity of healthcare work. Unlike fields such as sports, where flow often manifests as the automated execution of highly skilled abilities, healthcare professionals often need to handle multiple tasks simultaneously and respond to sudden emergencies. Medical practice requires practitioners to maintain constant awareness of risks and monitor situations while acting, in order to cope with dynamically changing clinical situations. This continuous risk awareness may hinder the full integration of action and awareness, but it does not mean a lack of flow experience. The core characteristics of flow, such as concentration, sense of control, transformation of time and loss of self-consciousness, are still fully preserved through other dimensions. Therefore, the lack of “Action-Awareness Merging” dimension did not weaken the construct validity of the scale, but instead revealed the unique integration characteristics of flow experience in the healthcare context, enhancing the situational adaptability of the scale.

In terms of practical significance, the scale developed in this study provides a preliminary tool for future exploration of the effects of management interventions in healthcare institutions. For example, by regularly assessing the flow level of healthcare workers, future research can examine whether it is effective to identify individuals at high risk of burnout who are in a long-term low flow state. The dimensional structure of the scale also provides theoretical insights for management practice. For example, the Concentration and Directional Feedback dimension highlights the importance of clear task goals and information feedback mechanisms; the Intrinsic Empowerment and Control dimension indicates that paying attention to the fit between work challenges and employees’ abilities, and avoiding keeping employees in a prolonged state of high pressure and low control, could be a potential direction for organizational intervention. However, it should be noted that the above practical implications are mainly based on the flow dimension structure of healthcare professionals and its theoretical connotations revealed in this study, representing a preliminary theoretical exploration. The scale’s predictive ability for important external variables such as burnout, work performance, and turnover intention needs to be further tested in future research. Once these empirical connections are established, this scale will provide more targeted management insights for healthcare institutions.

Although the developed flow state scale for healthcare professionals showed good reliability and validity and was validated based on a large sample (*n* = 1,240), there are still certain limitations. Firstly, regarding expert consultation and sample representativeness. The 15 experts participating in the Delphi consultation were all from the Beijing area, which may potentially affect the content validity of the scale on a national level. Although the core theoretical constructs of flow experience have been confirmed to have cross-cultural universality (Fournier et al., 2007; Kawabata et al., 2008), and the expert group members possess high heterogeneity and authority in their professional fields, the geographical concentration may still limit the full coverage of items in terms of the described scenarios across different regional medical work settings. The sample size in the pilot test was relatively small (n = 65) and non-probability sampling was adopted. Although participants covered different hospital levels and occupational categories, the sample size and sampling method somewhat limit the generalizability of the results and make it difficult to fully reflect the diversity of healthcare professionals. Future scale refinement studies should consider including larger and more representative pilot samples. The formal survey used an online convenience sampling strategy, which carries the risk of self-selection bias and online platform effects. Geographically, the sample was mainly concentrated in Beijing and Fujian, and primarily consisted of female participants, physicians, and staff from tertiary hospitals. These imbalances in composition may also limit the generalizability of the study results. Although we adopted stratified recruitment targets and multi-step data cleaning to mitigate these biases, their effects cannot be completely eliminated. Future research could use stratified probability sampling with broader geographic coverage to ensure a balanced sample across different regions, hospital levels, occupational categories, and genders. Secondly, regarding measurement methods and study design. The data came from a single self-reported questionnaire, which may involve common method bias. Healthcare professionals, as a group with high professional ethics, may be influenced by social desirability bias when completing the questionnaire. Although the anonymous design has alleviated this issue to some extent, it still requires verification through subsequent research incorporating multi-source data or behavioral indicators. Due to practical limitations such as the heavy clinical work and irregular shifts, the reliability of retesting has not been evaluated. Additionally, the study used a cross-sectional design and retrospective self-assessment questionnaire, which may have recall bias and cannot capture the dynamic changes in flow state. Future research could design feasible plans for retesting in more stable groups of healthcare workers, while also employing experience sampling methodology (Hektner et al., 2007) or other real-time data collection methods to collect information about the experiences of flow occurring at the moment of work. In addition, during the scale structure validation process, multiple revisions to the model based on statistical indicators may introduce the risk of construct drift. However, this study minimized this risk through the following design: ① Exploratory factor analysis and confirmatory factor analysis were conducted on two independent subsamples, respectively, and the final structure was well cross-validated in Sample 2. ② All deleted items were based on clear statistical criteria, and the model was reassessed after each adjustment. ③ During modifications, handling of MI was strictly limited within the same factor without introducing cross-factor correlations, thus preserving the theoretical purity of the structure. ④ The final four-factor structure has clear theoretical interpretability and aligns with the healthcare work context, indicating that no substantial construct drift occurred. Therefore, although model adjustments were inevitable, the stability and theoretical rationality of the final structure are supported. Future research can further verify the replicability of this structure in new independent samples. Finally, regarding validity testing and cross-group applicability. This study did not analyze the relationship between flow scores and external variables such as work performance and occupational burnout, lacking evidence of criterion and predictive validity. Moreover, cross-group invariance testing was not conducted, so the applicability of the scale to subgroups such as different genders, occupational categories, and hospital levels is unknown. Future research should supplement evidence of predictive validity and conduct cross-group invariance tests to verify whether the scale can be used in different roles or environments.

## 5 Conclusion

This study successfully developed and validated a flow state scale suitable for healthcare professionals. The four-dimensional scale with 15 questions has good reliability and validity, providing a reliable and effective tool for evaluating the flow experience of healthcare workers. By comprehensively assessing the flow states of healthcare workers, the scale can provide valuable reference for the managers of healthcare institutions in optimizing human resource management strategies (Kumar et al., 2025), enhancing employee motivation (Fullagar and Kelloway, 2009), and preventing occupational burnout (Aust et al., 2022). The application of this tool will also help to promote the transformation of healthcare organization management paradigm from the traditional problem orientation to the scientific mode of cultivating positive psychological capital and improving the overall work efficiency and service quality.

## Data Availability

The raw data supporting the conclusions of this article will be made available by the authors, without undue reservation.
