# Supplementary material for: Development and validation of a flow state scale for healthcare professionals
Source: Front Psychol. 2026 Apr 1;17:1751308. doi: 10.3389/fpsyg.2026.1751308 (PMC13079051; doi:10.3389/fpsyg.2026.1751308)
Supplement: Supplementary file 1 [file Table_1.DOCX]

Supplementary Material

1 Theory-Item Mapping Matrix: From Nine-Dimensional Flow to Healthcare-Contextualized Items

Table S1. Mapping of Theoretical Dimensions to Healthcare-Contextualized Items

| Theoretical Dimension | Operational Definition in Healthcare Context | Healthcare-Contextualized Items from Preliminary Pool |
| --- | --- | --- |
| **1. Challenge–Skill Balance** | Healthcare professionals believe that the challenges of current medical service tasks match their professional abilities, especially when both are at a high level. | • “When facing challenges in medical service, I believe my professional skills are sufficient to handle them.” • “My medical service skills matched the high demands of the current situation.” • “I felt my ability was enough to meet the high demands of the current medical service situation.” • “The medical service challenges and my professional skills were both at equally high levels.” |
| **2. Action–Awareness Merging** | Medical services feel automatic, intuitive, and occur without deliberate step-by-step reasoning; healthcare professional experiences a sense of "flowing" with the task. | • “My medical service actions seemed to occur automatically and naturally.” • “My medical service actions were performed subconsciously, without much thought.” • “My medical service actions were performed instinctively and habitually, requiring no conscious thought.” |
| **3. Clear Goals** | The healthcare professional has a clear understanding of the diagnostic, therapeutic, nursing, and other objectives for the current patient or medical task. | • “I knew clearly what I wanted to do.” • “I was clearly aware of my medical service duties and goals.” • “My goals for the medical service were clearly defined.” |
| **4. Unambiguous Feedback** | The practitioner receives immediate, clear information about their performance, typically through patient responses, vital signs, test results, or procedural outcomes. | • “I was indeed very clear about how I was performing in the medical service.” • “I knew how my medical service performance was.” • “I was well aware of how I performed while completing the medical service task.” • “I could judge my performance based on the medical service task I was completing.” |
| **5. Concentration on the Task at Hand** | Full attentional immersion in the medical service activities; irrelevant stimuli (environmental noise, personal concerns) are excluded from consciousness. | • “My attention was completely focused on the current medical service work.” • “I could concentrate on the current medical service work without any effort.” • “I was fully absorbed in every detail of the current medical service work.” • “I was fully immersed in the current medical service work.” |
| **6. Sense of Control** | The feeling that one can successfully manage the medical service activities and execute necessary procedures. | • “I felt that I could control the current medical service activities.” • “I felt completely in control of every aspect of the current medical service activity.” • “I felt I had complete control over my body to meet the needs of the medical service activity.” |
| **7. Loss of Self-Consciousness** | Absence of concern about how one is perceived by patients, colleagues, or supervisors; focus is entirely on the task, not on self-presentation. | • “I did not care how patients or colleagues viewed me.” • “I was not concerned about how others might evaluate my medical service behavior.”  • “I was not concerned about my self-presentation in medical services.” • “I was not worried about how patients or colleagues might view me.” |
| **8. Transformation of Time** | Subjective alteration of time perception during the medical service activity; time may pass faster than usual, slower than usual, or in a distorted manner. | • “During the medical service, time seemed to change (slower or faster).” • “During the current medical service activity, time passed differently than usual.” • “I lost my normal sense of time regarding the current medical service activity.” • “I felt that time passed more quickly during the medical service than usual.” |
| **9. Autotelic Experience** | Providing medical services is an inherent reward and enjoyment; the activity is undertaken for its own sake, not merely for external rewards. | • “I really enjoyed my medical service experience.” • “I loved the feeling of this medical service and wanted to experience it again.” • “The medical service process made me feel very good.” • “The experience of providing medical services to patients was the best reward.” |

Note: All items were rated on a 5-point Likert scale (1 = strongly disagree, 5 = strongly agree). Respondents were instructed to reflect on “the medical service activity you have just completed.”

**2 Preliminary flow state scale for healthcare professionals**

Please answer the following questions based on your experience during the medical service activity you have just completed. These questions relate to the thoughts and feelings you may have experienced during the process. There are no right or wrong answers. Reflect on how you felt during the medical service, and then respond using the rating scale provided. Please mark "√" under the number that best matches your experience.

| Classification | | | | |
| --- | --- | --- | --- | --- |
| Strongly Disagree | Disagree | Neither agree nor disagree | Agree | Strongly Agree |
| 1 | 2 | 3 | 4 | 5 |

1. When facing challenges in medical service, I believe my professional skills are sufficient to handle them.

1 - 2 - 3 - 4 - 5

1. My medical service actions seemed to occur automatically and naturally.

1 - 2 - 3 - 4 - 5

1. During the medical service, I knew clearly what I wanted to do.

1 - 2 - 3 - 4 - 5

1. I was indeed very clear about how I was performing in the medical service.

1 - 2 - 3 - 4 - 5

1. My attention was completely focused on the current medical service work.

1 - 2 - 3 - 4 - 5

1. I felt in control of my medical service activities.

1 - 2 - 3 - 4 - 5

1. During the medical service process, I did not care how patients or colleagues viewed me.

1 - 2 - 3 - 4 - 5

1. During the medical service, time seemed to change.

1 - 2 - 3 - 4 - 5

1. I greatly enjoyed the moments during the medical service.

1 - 2 - 3 - 4 - 5

1. My medical service skills matched the high challenges of the current situation.

1 - 2 - 3 - 4 - 5

1. My medical service actions were performed subconsciously, without much thought.

1 - 2 - 3 - 4 - 5

1. I was clearly aware of my medical service duties and goals.

1 - 2 - 3 - 4 - 5

1. I understood my performance in the medical service.

1 - 2 - 3 - 4 - 5

1. I could concentrate on the current medical service work without any effort.

1 - 2 - 3 - 4 - 5

1. I felt I could (or was able to) completely control every aspect of the current medical service activity.

1 - 2 - 3 - 4 - 5

1. During the medical service process, I was not concerned about how others might evaluate my medical service behavior.

1 - 2 - 3 - 4 - 5

1. During the current medical service activity, time passed differently than usual.

1 - 2 - 3 - 4 - 5

1. I loved this feeling of providing medical services and wanted to experience it again.

1 - 2 - 3 - 4 - 5

1. I felt my ability was sufficient to meet the high demands of the current medical service situation.

1 - 2 - 3 - 4 - 5

1. My medical service actions were habitual, requiring no conscious thought.

1 - 2 - 3 - 4 - 5

1. My goals for the medical service were clearly defined.

1 - 2 - 3 - 4 - 5

1. During the medical service task, I was fully aware of how I was performing.

1 - 2 - 3 - 4 - 5

1. I was fully absorbed in every detail of the current medical service work.

1 - 2 - 3 - 4 - 5

1. I felt I had complete control over my body to meet the needs of the medical service activity.

1 - 2 - 3 - 4 - 5

1. During the medical service process, I was not worried about how patients or colleagues might view me.

1 - 2 - 3 - 4 - 5

1. During the medical service, I lost the normal sense of time.

1 - 2 - 3 - 4 - 5

1. The medical service experience made me feel very good.

1 - 2 - 3 - 4 - 5

1. The medical service challenges and my professional skills were both at equally high levels.

1 - 2 - 3 - 4 - 5

1. I could judge my performance based on the medical service task I was completing.

1 - 2 - 3 - 4 - 5

1. I was fully immersed in the current medical service work.

1 - 2 - 3 - 4 - 5

1. I felt that time passed more quickly during the medical service than usual.

1 - 2 - 3 - 4 - 5

1. The experience of providing medical service to patients was the best form of encouragement.

1 - 2 - 3 - 4 - 5

**3 The flow state scale for healthcare professionals**

Please answer the following questions based on your experience during the medical service activity you just completed. These questions relate to various thoughts and feelings you might have experienced during the medical service process you just finished. There are no right or wrong answers. Think about how you felt during the medical service process, then use the scale below to answer. Mark "√" under the number that best matches your experience for each question.

| Classification | | | | |
| --- | --- | --- | --- | --- |
| Strongly Disagree | Disagree | Neither agree nor disagree | Agree | Strongly Agree |
| 1 | 2 | 3 | 4 | 5 |

1. I was indeed very clear about how I was performing in the medical service.

1 - 2 - 3 - 4 - 5

1. My attention was completely focused on the current medical service work.

1 - 2 - 3 - 4 - 5

1. I was clearly aware of my medical service duties and goals.

1 - 2 - 3 - 4 - 5

1. I understood my performance in the medical service.

1 - 2 - 3 - 4 - 5

1. My medical service skills matched the high challenges of the current situation.

1 - 2 - 3 - 4 - 5

1. I felt I could (or was able to) completely control every aspect of the current medical service activity.

1 - 2 - 3 - 4 - 5

1. I loved this feeling of providing medical services and want to experience it again.

1 - 2 - 3 - 4 - 5

1. I felt my ability was sufficient to meet the high demands of the current medical service situation.

1 - 2 - 3 - 4 - 5

1. The medical service challenges and my professional skills were both at equally high levels.

1 - 2 - 3 - 4 - 5

1. During the medical service, time seemed to change.

1 - 2 - 3 - 4 - 5

1. During the current medical service activity, time passed differently than usual.

1 - 2 - 3 - 4 - 5

1. I felt that time passed more quickly during the medical service than usual.

1 - 2 - 3 - 4 - 5

1. During the medical service process, I did not care how patients or colleagues viewed me.

1 - 2 - 3 - 4 - 5

1. During the medical service process, I was not concerned about how others might evaluate my medical service behavior.

1 - 2 - 3 - 4 - 5

1. During the medical service process, I was not worried about how patients or colleagues might view me.

1 - 2 - 3 - 4 - 5

(Note: The scale items are categorized by dimension, which are in order: Concentration and Directional Feedback, Intrinsic Empowerment and Control, Transformation of Time, Loss of Self-Consciousness.)

**4 Retention and Deletion of Initial Nine Flow Dimensions After CFA**

| Initial Nine Dimensions | Initial Item Content | Final Retained Item Content | Outcome Description |
| --- | --- | --- | --- |
| Challenge–Skill Balance | • “When facing challenges in medical service, I believe my professional skills are sufficient to handle them.”  • “My medical service skills matched the high challenges of the current situation.”  • “I felt my ability was sufficient to meet the high demands of the current medical service situation.”  • “The medical service challenges and my professional skills were both at equally high levels.” | • “My medical service skills matched the high challenges of the current situation.”  • “I felt my ability was sufficient to meet the high demands of the current medical service situation.”  • “The medical service challenges and my professional skills were both at equally high levels.” | Partially retained (3/4); items merged into "Intrinsic Empowerment and control" |
| Action–Awareness Merging | • “My medical service actions seemed to occur automatically and naturally.”  • “My medical service actions were performed subconsciously, without much thought.”  • “My medical service actions were habitual, requiring no conscious thought.” | 0 | Fully deleted (0/3) due to factor loadings < 0.5 in EFA |
| Clear Goals | • “During the medical service, I knew clearly what I wanted to do.”  • “I was clearly aware of my medical service duties and goals.”  • “My goals for the medical service were clearly defined.” | • “I was clearly aware of my medical service duties and goals.” | Partially retained (1/3); items merged into "Concentration and Directional Feedback" |
| Unambiguous Feedback | • “I was indeed very clear about how I was performing in the medical service.”  • “I understood my performance in the medical service.”  • “During the medical service task, I was fully aware of how I was performing.”  • “I could judge my performance based on the medical service task I was completing.” | • “I was indeed very clear about how I was performing in the medical service.”  • “I understood my performance in the medical service.” | Partially retained (2/4); items merged into "Concentration and Directional Feedback" |
| Concentration on the Task at Hand | • “My attention was completely focused on the current medical service work.”  • “I could concentrate on the current medical service work without any effort.  • “I was fully absorbed in every detail of the current medical service work.”  • “I was fully immersed in the current medical service work.” | • “My attention was completely focused on the current medical service work.” | Partially retained (1/4); items merged into "Concentration and Directional Feedback" |
| Sense of Control | • “I felt in control of my medical service activities.”  • “I felt I could (or was able to) completely control every aspect of the current medical service activity.”  • “I felt I had complete control over my body to meet the needs of the medical service activity.” | • “I felt I could (or was able to) completely control every aspect of the current medical service activity.” | Partially retained (1/3); items merged into "Intrinsic Empowerment and Control" |
| Loss of Self-Consciousness | • “During the medical service process, I did not care how patients or colleagues viewed me.”  • “During the medical service process, I was not concerned about how others might evaluate my medical service behavior.”  • “During the medical service process, I was not worried about how patients or colleagues might view me.” | • “During the medical service process, I did not care how patients or colleagues viewed me.”  • “During the medical service process, I was not concerned about how others might evaluate my medical service behavior.”  • “During the medical service process, I was not worried about how patients or colleagues might view me.” | Fully retained (3/3); forms independent dimension "Loss of Self-Consciousness" |
| Transformation of Time | • “During the medical service, time seemed to change.”  • “During the current medical service activity, time passed differently than usual.”  • “During the medical service, I lost the normal sense of time.”  • “I felt that time passed more quickly during the medical service than usual.” | • “During the medical service, time seemed to change.”  • “During the current medical service activity, time passed differently than usual.”  • “I felt that time passed more quickly during the medical service than usual.” | Mostly retained (3/4); forms independent dimension “Transformation of Time” |
| Autotelic Experience | • “I greatly enjoyed the moments during the medical service.”  • “I loved this feeling of providing medical services and wanted to experience it again.”  • “The medical service experience made me feel very good.”  • “The experience of providing medical service to patients was the best form of encouragement.” | • “I loved this feeling of providing medical services and wanted to experience it again.” | Partially retained (1/4); items merged into "Intrinsic Empowerment and Control" |

**5 Supplementary HTMT Examination and Confidence Intervals**

Table S2 Discriminant Validity (HTMT Ratio) for Sample 1

|  | Concentration and directional feedback | Intrinsic Empowerment and control | Transformation of time | Loss of self-consciousness |
| --- | --- | --- | --- | --- |
| Concentration and directional feedback |  |  |  |  |
| Intrinsic Empowerment and control | 0.769 |  |  |  |
| Transformation of time | 0.582 | 0.553 |  |  |
| Loss of self-consciousness | 0.477 | 0.728 | 0.456 |  |

Table S3 Bias-Corrected Confidence Intervals for HTMT in Sample 1

|  | Original sample (O) | Sample mean (M) | Bias | 5.0% | 95.0% |
| --- | --- | --- | --- | --- | --- |
| Intrinsic Empowerment and control -> Concentration and directional feedback | 0.769 | 0.769 | -0.000 | 0.709 | 0.823 |
| Loss of self-consciousness -> Concentration and directional feedback | 0.477 | 0.476 | -0.001 | 0.391 | 0.558 |
| Loss of self-consciousness -> Intrinsic Empowerment and control | 0.728 | 0.727 | -0.000 | 0.662 | 0.786 |
| Transformation of time -> Concentration and directional feedback | 0.582 | 0.580 | -0.001 | 0.487 | 0.672 |
| Transformation of time -> Intrinsic Empowerment and control | 0.553 | 0.552 | -0.001 | 0.453 | 0.639 |
| Transformation of time -> Loss of self-consciousness | 0.456 | 0.457 | 0.001 | 0.350 | 0.553 |

Table S4 Discriminant Validity (HTMT Ratio) for Sample 2

|  | Concentration and directional feedback | Intrinsic Empowerment and control | Loss of self-consciousness | Transformation of time |
| --- | --- | --- | --- | --- |
| Concentration and directional feedback |  |  |  |  |
| Intrinsic Empowerment and control | 0.807 |  |  |  |
| Loss of self-consciousness | 0.398 | 0.620 |  |  |
| Transformation of time | 0.693 | 0.715 | 0.547 |  |

Table S5 Bias-Corrected Confidence Intervals for HTMT in Sample 2

|  | Original sample (O) | Sample mean (M) | Bias | 5.0% | 95.0% |
| --- | --- | --- | --- | --- | --- |
| Intrinsic Empowerment and control -> Concentration and directional feedback | 0.807 | 0.808 | 0.002 | 0.731 | 0.857 |
| Loss of self-consciousness -> Concentration and directional feedback | 0.398 | 0.397 | -0.000 | 0.305 | 0.477 |
| Loss of self-consciousness -> Intrinsic Empowerment and control | 0.620 | 0.619 | -0.000 | 0.535 | 0.694 |
| Transformation of time -> Concentration and directional feedback | 0.693 | 0.695 | 0.002 | 0.569 | 0.792 |
| Transformation of time -> Intrinsic Empowerment and control | 0.715 | 0.721 | 0.005 | 0.557 | 0.839 |
| Transformation of time -> Loss of self-consciousness | 0.547 | 0.547 | 0.001 | 0.449 | 0.628 |

**6 Item Renumbering Mapping Table**

| New Item No. (Figure 3) | Original Item No. | Dimension | Item Wording |
| --- | --- | --- | --- |
| Q1 | Q4 | Concentration and Directional Feedback | I was indeed very clear about how I was performing in the medical service. |
| Q2 | Q5 | Concentration and Directional Feedback | My attention was completely focused on the current medical service work. |
| Q3 | Q12 | Concentration and Directional Feedback | I was clearly aware of my medical service duties and goals. |
| Q4 | Q13 | Concentration and Directional Feedback | I understood my performance in the medical service. |
| Q5 | Q10 | Intrinsic Empowerment and Control | My medical service skills matched the high challenges of the current situation. |
| Q6 | Q15 | Intrinsic Empowerment and Control | I felt I could (or was able to) completely control every aspect of the current medical service activity. |
| Q7 | Q18 | Intrinsic Empowerment and Control | I loved this feeling of providing medical services and wanted to experience it again. |
| Q8 | Q19 | Intrinsic Empowerment and Control | I felt my ability was sufficient to meet the high demands of the current medical service situation. |
| Q9 | Q28 | Intrinsic Empowerment and Control | The medical service challenges and my professional skills were both at equally high levels. |
| Q10 | Q8 | Transformation of Time | During the medical service, time seemed to change. |
| Q11 | Q17 | Transformation of Time | During the current medical service activity, time passed differently than usual. |
| Q12 | Q31 | Transformation of Time | I felt that time passed more quickly during the medical service than usual. |
| Q13 | Q7 | Loss of Self-Consciousness | During the medical service process, I did not care how patients or colleagues viewed me. |
| Q14 | Q16 | Loss of Self-Consciousness | During the medical service process, I was not concerned about how others might evaluate my medical service behavior. |
| Q15 | Q25 | Loss of Self-Consciousness | During the medical service process, I was not worried about how patients or colleagues might view me. |

**7 Competing Model Comparison Analysis**

To test the robustness of the four-factor structure, we conducted competing model comparisons using data from Sample 2b (n = 310).

Sample Split and Analytical Approach Description

This study employed a rigorous cross-validation design in sample splitting to ensure the independence of model validation and the reliability of results. Specifically, the total sample (N = 1240) was first randomly split into Sample 1 (n = 620) and Sample 2 (n = 620). Sample 1 was used for item analysis and initial reliability and validity testing to establish the factor structure, while Sample 2 (n = 620) was reserved for cross-validation. Sample 2 was further randomly divided into two subsamples: Sample 2a (n = 310) was used to replicate the factor structure through EFA to test the stability of the factor structure obtained from Sample 1, and Sample 2b (n = 310) was specifically reserved for confirmatory factor analysis (CFA) and competing model comparisons. Competing model comparisons fall within the domain of confirmatory analysis, belonging to the same category as CFA. Therefore, conducting them on the same validation sample (Sample 2b) is appropriate and does not introduce "data snooping" bias. It is important to emphasize that both CFA and competing model comparisons were performed using the raw, unmodified Sample 2b data, and all analyses were based on the final set of 15 items (item deletion was completed in Sample 1, and Sample 2b was used solely for validation). During the CFA process, we only examined model fit and did not make any adjustments to the model (e.g., adding paths or deleting items based on modification indices), thereby ensuring the impartiality of the competing model comparisons and the reliability of the results.

Competing Model Specification

Single-factor model: All 15 items were forced to load onto a single latent factor (flow)；Two-factor model: Based on theoretical relevance, "Concentration and Directional Feedback" and "Intrinsic Empowerment and Control" were combined into one factor, while "Transformation of Time" and "Loss of Self-Consciousness" were combined into another；Three-factor model: The first two dimensions were merged, while "Transformation of Time" and "Loss of Self-Consciousness" were kept independent；Second-order factor model: Based on the four-factor model, a higher-order "flow" factor was specified to account for the covariance among the four first-order factors.

The results of the confirmatory factor analyses indicated that our proposed four-factor model (baseline model) demonstrated better fit across all indices compared to all competing models (see Table S6). This series of comparisons provides strong statistical support for the construct validity of the scale, confirming the reasonableness and superiority of the four-factor structure among healthcare professionals.

Table S6: Comparison of Fit Indices for Competing Models

| Model | χ²/df | RMSEA | CFI | TLI | SRMR | RMR | GFI | AGFI | AIC |
| --- | --- | --- | --- | --- | --- | --- | --- | --- | --- |
| Single-factor model | 10.640 | 0.177 | 0.742 | 0.699 | 0.102 | 0.115 | 0.659 | 0.546 | 1017.642 |
| Two-factor model | 8.511 | 0.156 | 0.801 | 0.765 | 0.087 | 0.093 | 0.691 | 0.583 | 819.472 |
| Three-factor model | 5.432 | 0.120 | 0.885 | 0.861 | 0.068 | 0.054 | 0.790 | 0.711 | 538.566 |
| Four-factor model | 2.661 | 0.073 | 0.958 | 0.948 | 0.049 | 0.037 | 0.915 | 0.878 | 295.498 |
| Second-order factor model | 2.941 | 0.079 | 0.950 | 0.939 | 0.058 | 0.047 | 0.905 | 0.867 | 320.923 |
